# Clinical outcomes and anesthetic management of pregnancies with placenta previa and suspicion for placenta accreta undergoing intraoperative abdominal aortic balloon occlusion during cesarean section

**DOI:** 10.1186/s12871-020-01040-8

**Published:** 2020-05-30

**Authors:** Peng Li, Xia Liu, Xiangkui Li, Xinchuan Wei, Juan Liao

**Affiliations:** 1 Department of anesthesiology, Sichuan Academy of Medical Sciences & Sichuan Provincial People’s Hospital, Chengdu, Sichuan China; 2 North Sichuan Medical College, Nanchong, Sichuan China; 3 Department of Stomatology, Sichuan Academy of Medical Sciences & Sichuan Provincial People’s Hospital, Chengdu, Sichuan China

**Keywords:** Placenta previa, Placenta accreta, Cesarean section, Intraoperative abdominal aortic balloon occlusion, Anesthetic management

## Abstract

**Background:**

This retrospective study aimed to compare the clinical outcomes of parturients with placenta previa (PP) and placenta accreta (PA) according to their severity, when they were managed with intraoperative abdominal aortic balloon occlusion (IAABO) during cesarean section.

**Methods:**

We retrospectively examined 57 cases of PP and suspicion for PA in which IAABO was performed during cesarean section between April 2014 and June 2016. Based on preoperative examination and clinical risk factors, patients were divided into the low suspicion PA group and the high suspicion PA group. We compared the demographic characteristics, methods of anesthesia, intra- and postoperative parameters, and maternal and neonatal outcomes.

**Results:**

The two groups showed similar demographic characteristics and intraoperative outcomes. Four women underwent cesarean hysterectomy. Eight neonates were admitted to the neonatal intensive care unit and three did not survive. Neonatal Apgar scores were significantly higher in the low suspicion PA group. Eight patients experienced postoperative femoral artery thrombosis and one patient complicated hematoma in the front wall of the common femoral artery. Patients who received neuraxial anesthesia showed significantly lower intraoperative blood loss, lower intraoperative, postoperative and total blood transfusion and shorter surgery than patients who received general anesthesia.

**Conclusions:**

Our data suggested that the severity of aberrant placental position does not affect intraoperative blood loss during a cesarean section while the IAABO is performed. We propose that neuraxial anesthesia is preferred for conducting these surgeries without contraindications.

## Background

Placenta accreta spectrum, composed of placenta accreta, increta, and percreta, can result in severe hemorrhage, and lead to significant maternal morbidity and mortality rates [1, 2]. In fact, the term “accreta” was viewed as a common term for the above three conditions. Risk factors for abnormally invasive placentation include placenta previa with or without previous uterine surgery, prior myomectomy, prior cesarean delivery, Asherman’s syndrome, submucous leiomyomata and maternal age older than 35 years [3]. The rates of PP and PA are increasing due to the rising incidence of cesarean section [3, 4]. Careful management of the anesthetic protocol is critical for women with placenta previa who have a history of cesarean section or abnormally invasive placentation [5].

IAABO has been extensively used in major pelvic surgical procedures and is effective in reducing intraoperative hemorrhage [6]. Moreover, this intravascular interventional therapy has been shown to effectively reduce intraoperative hemorrhage in patients with placenta accreta [7, 8]. Therefore, this technique is performed in our hospital to control severe intraoperative hemorrhages in patients with PP and suspicion for PA. However, not much is known about the best anesthetic protocol to choose when dealing with this situation. Therefore, this retrospective study was performed to compare the clinical outcomes of parturients with placenta previa and placenta accreta according to their severity, when they were managed with IAABO during cesarean section. In addition, we also evaluated the influence of the anesthesia method on postoperative clinical outcomes in this population.

## Methods

### Subjects

This retrospective study included 57 pregnancies between April 2014 and June 2016 at the Sichuan Provincial People’s Hospital (Chengdu, China). This study was approved by the Ethics Committees of the Sichuan Academy of Medical Sciences and Sichuan Provincial People’s Hospital and consent was waived.

We included patients with placenta previa and suspicion for placenta accreta who opted for IAABO during their cesarean section. Whether to perform the IAABO on patients was actually depended on the surgeons’ synthetic judgment and patients’ will. The aortic catheterization was performed on patients by interventional radiology preoperatively who were diagnosed with PP and suspected of having placenta accreta. Before the surgeon made an incision in the serous membrane of the uterus, according to the surgeon’s request, sterilizing saline was injected into the balloon to control bleeding. The volume of the sterilizing saline was based on the situation of preoperative intervention, which demonstrated a satisfactory outcome of interfering with blood flow. After the placenta was extracted without active bleeding, the balloon was deflated by drawing saline out slowly. The duration of IAABO was less than 60 min at one time. If the surgeons wanted to occlude blood flow again, there should be at least 10-15 min periods of intermittence to restore blood flow. The catheters were usually extracted by the radiologists when the pregnancies’ vital signs were stable after surgery.

The subjects in our study were divided into a low suspicion PA group and a high suspicion PA group conducted by two specialistic obstetricians on the basis of preoperative diagnosis by ultrasound or MRI (magnetic resonance imaging) and clinical risk factors [9]. High suspicion PA group comprised cases meeting the following criteria: (1) high suspicion for PA according to ultrasonography or MRI imaging findings including deficiency of retroplacental sonolucent zone; segmental retroplacental myometrial thinning < 1 mm; multiple vascular lacunae presenting a ‘moth hole’ appearance [10]; (2) pernicious placenta previa defined as PP with anterior placenta overlying a previous scar. In addition, low suspicion PA group included cases: (1) low-suspicion ultrasound for PA and anterior placenta without history of caesarean surgery; (2) PP without ultrasound signs for PA but with one previous caesarean section [9].

The following information was recorded: age, parity, gestational age, history of surgical abortion, previous cesarean section, type of anesthesia, intraoperative blood loss, blood transfusion, neonatal Apgar scores, duration of surgery and postoperative complications. After data collection, subjects were further stratified into a neuraxial anesthesia group or a general anesthesia group.

### Statistical Analysi*s*

Statistical analysis was carried out using SPSS Statistics 24.0 (IBM, Chicago, IL, USA). Data were presented as mean ± SD or median (range). Intergroup differences were assessed for significance using Student’s *t*, Kruskal-Wallis, chi-squared or Fisher exact tests, as appropriate. *P* < 0.05 was considered statistically significant.

## Results

**Fig. 1:**
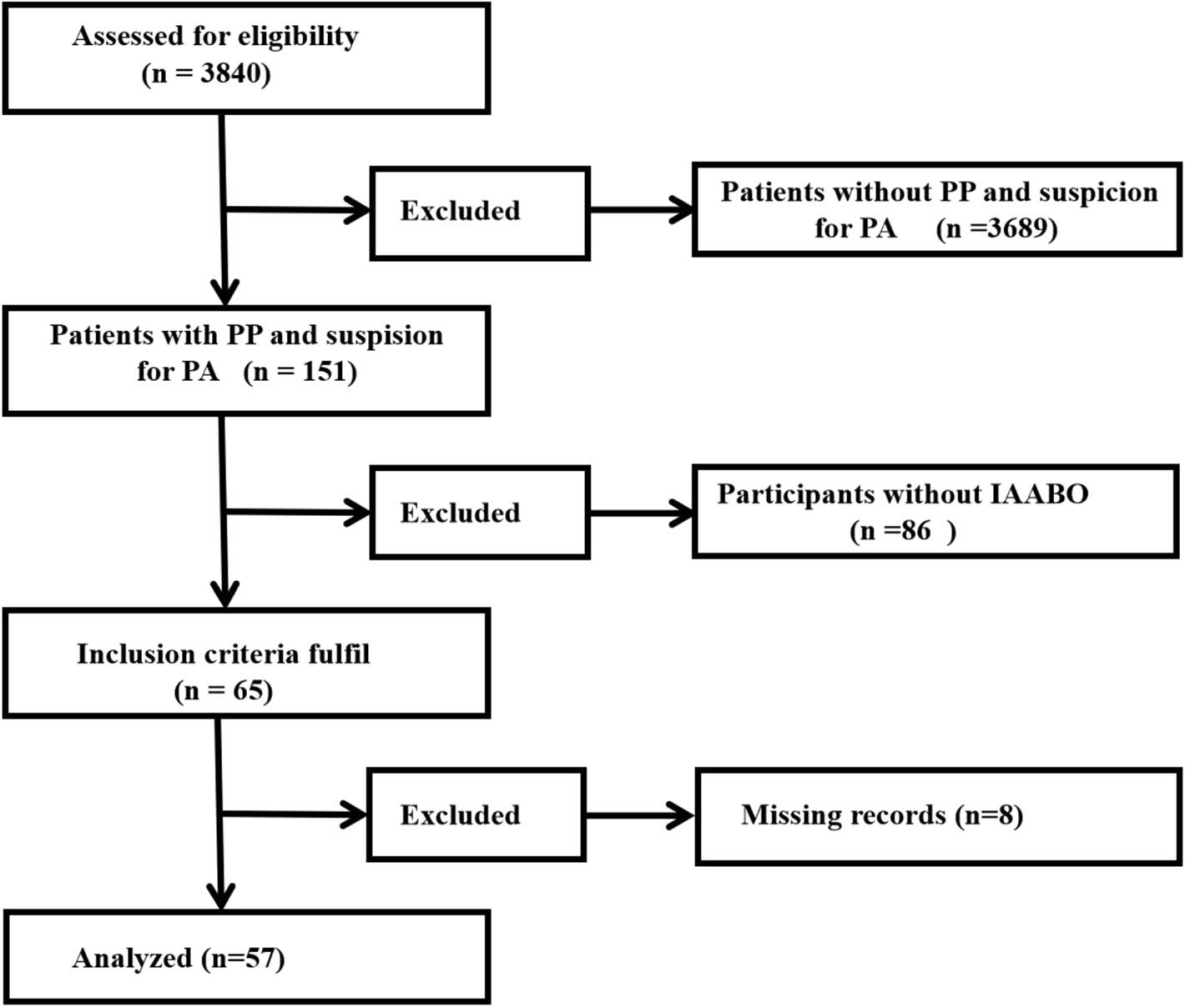
Flow chart of the trial

As listed in Fig. 1, a total of 3840 pregnant women underwent cesarean section between April 2014 and June 2016 in our hospital. After excluding cases whose preoperative diagnosis was not PP and suspicion for PA and without use of IAABO, we included 65 pregnant women meeting the inclusion criteria. Also, there were 8 patients without detailed recording. Thus, our study totally included 57 subjects for analysis. In our study, we finally made sure 32 pregnancies diagnosed with PA. The incidence of PA was found to be 0.83% (32/3840) of all pregnancies contemporarily. During the same period, the rate of utilization of the technique of IAABO was 43% (65/151) among the patients diagnosed with placenta previa and suspicion for placenta accreta.

A total of 57 pregnant patients were included in this study, of whom 27(47.3%) were older than 35 years. The sites of placenta attached to the uterus were anterior wall (*n* = 32,56%), posterior wall (*n* = 20, 35%) and lateral wall(*n* = 5, 9%). Across all deliveries, gestational age was  248 ± 20.8 days and 48 (84.2%) required emergency surgery. Reports on previous history revealed that the median and range of surgical abortion for all patients was 2 (0–6) with 3 (0–7) for uterine surgery (Table 1). Patients were divided into low suspicion or high suspicion for PA groups based on preoperative examination and clinical risk factors. Although the high suspicion PA group was more of the history of surgical abortion, cesarean section or uterine surgery than the low suspicion PA group, there was no difference of statistics.

**Table 1:** Baseline characteristics for patients with abnormal placenta position

Characteristic	Total (*n* = 57)	Low suspicion group (*n* = 10)	High suspicion group (*n* = 47)	*P*
Preoperative comorbidity	14 (24.6%)	5 (50.0%)	9 (19.1%)	0.098
Preoperative hemoglobin	107 ± 13.7	104 ± 12.4	107 ± 14.0	0.457
Emergency surgery	48 (84.2%)	9 (90.0%)	39 (83.0%)	0.940
Gestational age (days)	248 ± 20.8	243 ± 19.6	249 ± 21.0	0.353
Patient age (years)	36 ± 7.9	37 ± 8.9	36 ± 7.7	0.749
Parity	4 (1–8)	2.5 (2–8)	4 (1–8)	0.169
Previous surgical abortions	2 (0–6)	1 (0–6)	2 (0–6)	0.121
Previous cesarean sections	1 (0–2)	0 (0–1)	1 (0–2)	0.073
previous uterine surgeries	3 (0–7)	1 (0–7)	3 (0–7)	0.064

Values are n (%), mean ± SD or median (range)

Post-surgery data showed neonatal Apgar scores were higher in the low suspicion PA group compared to scores reported for the high suspicion PA group. Eight (17.0%) neonates from the high suspicion PA group were admitted to the neonatal intensive care unit, compared to none of the neonates from the low suspicion PA group. In the high suspicion PA group, there were three twin pregnancies, one of which resulted in the death of both babies after the family refused admission to the NICU (neonatal intensive care unit) and left the hospital against medical advice. One patient from the low suspicion PA group and 6 (12.8%) from the high suspicion PA group were moved into the ICU (intensive care unit). Four patients were admitted to ICU as a result of the greater intraoperative blood loss (≥2000 ml) and other 3 patients mainly were respectively on account of postoperative loss of consciousness, twin pregnancy combined with severe preeclampsia preoperatively and paroxysmal supraventricular tachycardia operatively. These differences in rates of admission to the neonatal or standard intensive care units were not statistically significant (Table 2).

**Table 2:** Maternal and neonatal intra- and postoperative outcomes for low and high suspicion for PA patients

Outcome	Low suspicion group (*n* = 10)	High suspicion group (*n* = 47)	*P*^a^
**Intraoperative data**			
Intraoperative blood loss (ml)	500 (200–800)	500 (200–3500)	0.784
Intraoperative blood transfusion (U)	0 (0–2)	0 (0–14)	0.818
Duration of surgery (min)	62 ± 10.0	79 ± 44.4	0.239
Intrauterine balloon tamponade	2 (20%)	1 (2.1%)	0.076
Uterine artery embolization	1 (10%)	8 (17.0%)	1.000
Hysterectomy	0	4 (8.5%)	1.000
**Postoperative complications**			
Femoral artery thrombosis	0	8 (17.0%)	1.000
Anesthesia-related complications	0	1 (2.1%)	1.000
Operation-related complications	1 (10%)	2 (4.3%)	1.000
**Postoperative data**			
Postoperative hospital stay (day)	5.5 ± 1.4	5.2 ± 2.7	0.745
Hemoglobin on postoperative day 1	106 ± 12.7	98 ± 15.1	0.139
Admission to ICU	1 (10%)	6 (12.8%)	1.000
**Postoperative diagnosis with PA**	2 (20%)	30 (64%)	0.016
Placenta accreta	1	14	
Placenta increta	1	10	
Placenta percreta	0	6	
**Postoperative clotting variables**			
Platelets(10^9^/L)	166 (95–333)	171 (71–328)	0.826
INR	0.92 (0.81–1.12)	0.93 (0.79–1.28)	0.842
PT(s)	10.8 (10.5–13.5)	10.5 (9.2–12.4)	0.122
**Neonatal data**			
Neonate Apgar score			
1 min	10 ± 0.0	8.9 ± 2.0	0.001
5 min	10 ± 0.0	9.5 ± 1.0	0.003
10 min	10 ± 0.0	9.7 ± 0.7	0.004
Weight (g)	2751 ± 491.6	2958 ± 470.2	0.237
Admission to NICU	0	8 (17.0%)	0.327

Values are n (%), mean ± SD or median (range) or median (range)

After abdominal aortic balloon occlusion during cesarean section, we found no significant differences in intraoperative blood loss, intraoperative blood transfusion, duration of surgery and postoperative hospital stay between the two groups. Because of bleeding during periods of intermittence of IAABO, the intrauterine balloon tamponade was used in 2 (20%) patients in the low suspicion PA group and 1 (2.1%) in the high suspicion PA group. Besides, the patients performed uterine artery embolization were 1 (10%) and 8 (17.0%) in each group respectively. Though there were no statistical difference with regard to clotting variables postoperatively between two groups, the intervention of the balloon block led to femoral artery thrombosis in 8 (17.0%) and local hematoma of the common femoral artery in 1 (4.3%) from the high suspicion PA group. Six patients underwent arteriotomy of the femoral artery plus embolectomy and two patients used low molecular heparin for anticoagulation. The patient complicated local hematoma refused further examination and the pain in her leg was obviously relieved in the postoperative day 3.

It is worth noting that 4 (8.5%) patients from the high suspicion PA group, but no one from the low suspicion PA group, underwent hysterectomy because of hemorrhaging and difficulties related to placenta dissection during surgery. The placentas of another 53 patients were dissected by hand or surgical instruments as much as possible without leaving placenta in situ. One patient in the high suspicion PA group also experienced anesthesia-related complications. The patient suffered postoperative nausea, vomiting, pain and diminished consciousness and then treated by mechanical ventilation for 2 days.

Postoperative operation-related complications were observed in three patients. A uterine artery embolization was performed on one patient in the low suspicion PA group in order to stop bleeding after the gauze was removed from the vagina. One subject from the high suspicion PA group experienced a pelvic hematoma, disturbance of blood coagulation and was treated with an abdominal laparotomy and received 1.5 units plasma transfusion. Another high suspicion for PA patient underwent cystourethroscopy for the evacuation of a cystic hematoma and was subsequently diagnosed with placenta percreta. Operative blood loss was 3500 ml and the patient received operative blood transfusion containing 10 units red blood cells, 4 units fresh frozen plasma and 10 units of cryoprecipitate. The patient remained in the hospital for 14 days, of which 3 were spent postoperatively in the intensive care unit without ventilation. No maternal mortality was reported. Placenta accreta was definitively diagnosed postoperatively based on pathology examination or surgeons’ classification when separating the placenta. Based on postoperative diagnosis, the low suspicion PA group actually contained 2 (20%) patients with placenta accreta, compared to 30 (64%) patients in the high suspicion PA group (Table 2).

To determine the effect of anesthesia on maternal and neonatal outcomes, patients were stratified based on whether they received neuraxial anesthesia (*n* = 43) or general anesthesia (*n* = 14). Thirteen (30.2%) patients underwent the subarachnoid anesthesia (L3–4) with injection 0.5% bupivacaine 2 ml. Another 30 patients with spinal-epidural anesthesia (L3–4) were injected the same local anesthetic without additional supplementary for analgesia. The induction drugs for general anesthesia mostly were propofol (2–2.5 mg.kg^− 1^), remifentanil (1.0 μg.kg^− 1^) and succinylcholine (1–1.5 mg.kg^− 1^) or rocuronium (0.6 mg.kg^− 1^). The anesthesia was maintained with remifentanil (0.2–0.25 μg.kg^− 1^ min^− 1^), propofol (4–6 mg.kg^− 1^ h^− 1^) and sevenflurane less than 1 MAC (minimum alveolar concentration). After the baby was took out, sufentanil (0.3–0.4 μg.kg^− 1^) and midazolam (0.03–0.05 mg.kg^− 1^) were injected to deepen anesthesia. The neuraxial anesthesia group comprised 8 patients from the low suspicion PA group and 35 from the high suspicion PA group; general anesthesia, 2 and 12. One patient firstly received regional anesthesia and then was switched to general anesthesia by anesthesiology team for the following concerns: history of thalassemia and severe preeclampsia, extended time of surgery, twin pregnancy, massive hemorrhage and hemodynamic instability. The two anesthesia groups showed significant differences in intraoperative blood loss, intraoperative blood transfusion, postoperative blood transfusion, total blood transfusion and duration of surgery (Table 3). The general anesthesia group tended to suffer greater blood loss and require more transfusion, longer surgery and more venous puncture.

**Table 3:** Comparison of clinical data between pregnancies involving neuraxial or general anesthesia

	Neuraxial anesthesia (*n* = 43)	General anesthesia (*n* = 14)	*P*^a^
**Baseline characteristics**			
Gestational age (days)	250 ± 18.4	252 ± 18.1	0.583
Patient age (years)	35 ± 7.8	38 ± 7.7	0.246
Emergency surgery	36 (83.7%)	12 (85.7%)	1.000
Preoperative comorbidity	9 (20.9%)	5 (35.7%)	0.271
CVC	1 (2.3%)	3 (21.4%)	0.042
Arterial catheterization	3 (7.0%)	4 (28.6%)	0.054
**Intraoperative data**			
Use of vasoactive agents	7 (16.3%)	2 (14.3%)	1.000
Intraoperative blood loss (ml)	500 (200–2000)	700 (300–3500)	0.017
Intraoperative blood infusion (U)	0 (0–4)	1.5 (0–14)	0.018
Duration of surgery (min)	65.7 ± 16.4	107.5 ± 70.4	0.046
**Postoperative data**			
Postoperative blood infusion (U)	0 (0–4)	0 (0–15.5)	0.001
Total blood infusion (U)	0 (0–6)	2.625 (0–29.5)	0.009
Maternal admission to ICU	3 (7.0%)	4 (28.6%)	0.054
Postoperative hospital stay (days)	4.9 ± 1.5	6.6 ± 4.2	0.146
Anesthesia-related complications	0	1 (7.1%)	0.246
**Postoperative diagnosis with PA**	23 (53.5%)	9 (64.3%)	0.479
Placenta accreta	13	2	
Placenta increta	8	3	
Placenta percreta	2	4	
**Neonatal data**			
Neonatal Apgar scores			
1 min	9.2 ± 1.75	8.9 ± 2.3	0.539
5 min	9.6 ± 0.9	9.5 ± 1.2	0.554
10 min	9.7 ± 0.7	9.7 ± 0.8	0.834
Neonatal admission to ICU	5 (11.6%)	3 (21.4%)	0.391

Values are n (%), mean ± SD or median (range)

## Discussion

Anesthetic management plays a significant role in ensuring the safety of patients with placenta previa and/or accreta. Factors contributing to the abnormally invasive placenta should be identified prior to medical intervention. Anesthetists should then take these risk factors into account and collaborate with other healthcare professionals to aid in planning the most appropriate anesthesia plan.

A review of 62 placenta accreta cases found that 73% of patients had at least one previous cesarean delivery, and 45% were older than 35 years [11]. Our study found a higher proportion of patients who were older than 35 years (47.3%), which could explain the increased ratio of surgical abortion (82.5%). The incidence of cesarean hysterectomy in our study (7%) was much lower than the range of 48–100% in previous studies [12–15]. As some studies demonstrated, IAABO might be helpful in decreasing the likelihood of hysterectomy [7, 16–18]. We did not observe any maternal mortalities, in contrast, a previous study reported maternal deaths in the absence of interventional operation [5]. Further research is required to determine whether the technique of IAABO could consistently reduce the rate of maternal mortalities. Some researchers may be afraid that the radiation originated from preoperative intervention may cause fetal damage. However, the International Commission on Radiological Protection (ICRP) suggested that the fetal teratogenic risk does not increase when the radiation dose is less than 100 mGy [19]. Besides, the article reported fetal radiation exposure doses resulting from the technique of IAABO were 4.9 ± 2.9 mGy [20]. Three other articles compared IAABO and bilateral internal iliac artery balloon occlusion in terms of the radiation dose and concluded that the former resulted in a lower fetal radiation dose [21–23]. Therefore, just like articles claimed the prophylactic use of abdominal aortic balloon occlusion in patients with placenta accreta is safe and effective [7, 8, 24].

Our study found no significant differences between the low and high suspicion PA groups in intraoperative blood loss and transfusion, postoperative hospitalization or rate of admittance to the ICU. These results suggest that the severity of aberrant placental position do not compromise the ability of IAABO to control severe hemorrhage. For example, we found an estimated median blood loss of 500 ml for the low and high suspicion PA groups, and this loss ranged from 200 to 3500 ml across the two groups. This was much lower than for patients who did not undergo such catheterization in previous studies [4, 24]. This variation in blood loss may be related to the technique of IAABO [16, 24]. When the 57 pregnancies diagnosed with PP and suspicion for PA based on preoperative examination (ultrasound or MRI) and clinical risk factors, the decision of using IAABO conducted by a multidisciplinary team (anesthesiologist, obstetrician, interventional radiologist and neonatologist). However, we should note that our study actually contained 32 patients with placenta accreta (accreta 15, increta 11, percreta 6) based on postoperative diagnosis. In fact, there were other 25 patients finally diagnosed with PP but with IAABO. Based on preoperative examinations (ultrasound, MRI) and clinical signs, 56.1% (32/57) of the patients suspicion for PA were identified correctly. Though there was still lack of definitive preoperative diagnostic tool, we should be well prepared for patients suspected of PA (even in low suspicion patients) because of high risk for massive bleeding. Meanwhile, we should pay more caution for its intervention-related complications. Altogether 8 (14.0%) of the patients in our study suffered from femoral artery thrombosis and some of them required femoral artery embolectomy. This catheterization-related complication may be due to the length of occlusion. According to literature, the best safety outcomes for IAABO occur when total occlusion time is less than 60 min [25]. Since the IAABO time was not included in medical records, we could not examine the effect of occlusion time on postoperative thrombosis in our study. Future studies should pay more attention to this. However, we could suppose that the occlusion time was shorter in the low suspicion PA. Moreover, Patients with the risk of femoral artery thrombosis was lower in the low suspicion PA group than the high suspicion PA group based on duration of surgery (62 ± 10.0 vs 79 ± 44.4), which needed to further investigation.

In this retrospective study, neuraxial anesthesia was used in 8 (80%) of the low suspicion PA pregnancies and 35 (74.5%) of the high suspicion PA ones, while only 2 (20%) patients of the low suspicion PA group and 12 (25.5%) of the high suspicion PA group performed general anesthesia. Final diagnosis with placenta accreta included totally 32 cases (accreta 15, increta 11, percreta 6), of whom 23 (71.9%) patients performed neuraxial anesthesia. The above results suggested that neuraxial anesthesia was used more often than general anesthesia for intraoperative aortic balloon occlusion intervention during a cesarean section. In contrast, one study found that general anesthesia was preferred during treatment of placenta previa, in order to avoid the risk of bleeding [5]. Another study indicated that general anesthesia was used almost exclusively for women strongly suspected of having placenta accreta, while spinal anesthesia was used in nearly two-thirds of cases with placenta previa without suspicion of placenta accreta [26]. These differences in anesthetic protocols may be attributed to the fact that we enrolled only patients who underwent intraoperative abdominal aortic balloon occlusion during a cesarean section.

As argued by Guasch, general anesthesia was a risk factor for transfusion [27]. In our study, we also found more perioperative blood loss, transfusion and duration of surgery in the general anesthesia group than that in the neuraxial anesthesia group. However, in this retrospective study, the parturients’ underlying conditions cannot be guaranteed to be absolutely consistent. The more blood loss and transfusion in the general anesthesia group could not completely exclude the influence of parturients’ coagulation disorders. Besides, there was one patient whose anesthetic method was switched from neuraxial anesthesia to general anesthesia due to massive hemorrhage intraoperatively. Thus, the general anesthesia group could be biased towards a potentially high risk of bleeding, which could be the limitation of this study.

The anesthesia methods for pregnant women with placenta previa (PP) and suspicion for placenta accreta (PA) were determined by many factors. For example, the will of patients’, preoperative comorbidities, coagulation conditions, duration of surgery, fetal condition, the risk for massive hemorrhage were all taken into account by a multidisciplinary team (anesthesiologist, obstetrician, interventional radiologist and neonatologist). There are some important risk factors for general anesthesia including coagulation disorders, massive bleeding in the third trimester, sever hemodynamic instability, fetal distress and severe pre-eclampsia [27]. However, faced with patients with placenta previa and suspicion for placenta accreta undergoing IAABO, neuraxial anesthesia may be preferable in our center excepting contraindications (coagulation disorders, infection of insertion point, sever lumbar spinal stenosis, hypovolemic shock and so on). This conclusion can be consistent with some previous studies, which concluded that neuraxial anesthesia is now employed more frequently for pregnancies with placenta accreta [12, 28]. The benefit of neuraxial anesthesia may include: (1) improvement of postoperative analgesia by PCEA (patient-controlled epidural analgesia);(2) related to less blood loss than general anesthesia at the time of hysterectomy [29]; (3) offered as an adjuvant to prevent thrombosis in high-risk patients [30]; and associated with lower incidence of postoperative thrombosis [31]; (4) minimization of the risk of failed intubation, ventilation and aspiration [32].

Only a small percentage of patients received arterial lines (12.3%) or central venous catheters (7%), which are lower than percentages reported in other studies [5, 26, 33]. We suspect the lower rate of invasive puncture is due to the expected decrease in bleeding after IAABO, but these patients should nevertheless be closely monitored.

In conclusion, the management of patients diagnosed with placenta previa and suspicion for placenta accreta requires a multidisciplinary approach. The anesthesiologist, obstetrician and interventional radiologist should formulate a plan to safely handle a massive hemorrhage that may occur during surgery. Prophylactic use of an abdominal aorta balloon catheterization may reduce the rate of cesarean hysterectomy and maternal mortality but needs further evidenced-based research to validate. Due to the potential advantages of neuraxial anesthesia, we prefer for this type of anesthesia in the absence of contraindications during abdominal aorta balloon catheterization intervention when treating patients diagnosed as placenta previa and suspicion for placenta accreta. Clinicians should, however, be aware of complications arising from the intraoperative abdominal aortic balloon occlusion.

## Data Availability

All data generated or analyzed during this study are included in this published article and supporting data can be obtained from the corresponding author.

## Abbreviations

PP: Placenta previa
PA: Placenta accreta
IAABO: Intraoperative abdominal aortic balloon occlusion
NICU: Neonatal intensive care unit
ICU: Intensive care unit
INR: International normalized ratio
PT: Prothrombin time
CVC: Central venipuncture catheterization
